# What are they considering when they face a fetus with birth defects? A qualitative study on ethical attitudes of health professionals in China

**DOI:** 10.1186/s41256-024-00370-1

**Published:** 2024-07-15

**Authors:** Yanlin Liu, Xiaomin Wang, Junqun Fang, Wei Zhou, Dan Luo

**Affiliations:** 1 Department of Social Medicine and Health Management, Xiangya School of Public Health, Central South University, Changsha, Hunan China; 2 School of Health Sciences, College of Medicine, Nursing and Health Sciences, University of Galway, Galway, Ireland; 3 Center for Clinical Pharmacology, The Third Xiangya Hospital of Central South University, Changsha, Hunan China; 4 Center of Medical Ethics, Central South University, Changsha, China; 5 Hunan Provincial Hospital of Maternal and Child Health Care, Changsha, Hunan China; 6 Research Center for Public Health and Social Security, School of Public Administration, Hunan University, Changsha, China

**Keywords:** Birth defects, China, Health professionals, Perspectives, Qualitative study

## Abstract

**Background:**

Birth defects are the leading cause of mortality in newborn babies and children under five years old. In response, the Chinese government has implemented a three-tiered prevention strategy, which has brought ethical concerns about fetuses with birth defects. This study aims to explore the attitudes toward fetuses with birth defects among health professionals engaged in maternal and child health services.

**Methods:**

A qualitative study was conducted among 13 health professionals engaged in maternal and child health services in Hunan Province, China. The questions were designed to elicit the participants' work experience and attitudes toward fetuses with birth defects. The data were collected through in-depth semi-structured interviews, and NVivo 12 was used for data coding and analysis. A thematic analysis approach was employed following the SRQR checklist.

**Results:**

Five themes and 13 attributes were generated regarding health professionals' perspectives on fetuses with birth defects. The five themes included: (1) severity and curability of diseases (two attributes), (2) family relations (four attributes), (3) medical assessments (two attributes), (4) social situations (three attributes), (5) self-value orientations (three attributes). The findings showed that the majority of health professionals held the view that a fetus with a curable disease could be born, whereas a fetus with severe disability and teratogenesis should be terminated. Twelve out of the 13 health professionals believed that parents should be the decision-makers, while only one thought that the family should make a decision together.

**Conclusions:**

Attitudes toward birth defects were influenced by various factors, indicating the complexity of real-world cases identified in this study. The findings highlight the dilemmas faced by both families and health professionals regarding birth defects. Adequate medical knowledge and support from society are crucial to inform decision-making among family members. Additionally, standardized norms and policies for birth defects are needed. Establishing an ethics committee for prenatal diagnosis is necessary to address current ethical issues in this field.

**Supplementary Information:**

The online version contains supplementary material available at 10.1186/s41256-024-00370-1.

## Background

Birth defects include structural or functional anomalies and metabolic disorders that occur during intrauterine life. These conditions develop prenatally and may be identified before birth, at birth, or later in life [1]. Each year, an estimated 6% of babies worldwide are born with birth defects, resulting in approximately 295,000 newborn deaths within the first four weeks of life. Birth defects are also a major cause of child mortality in the first five years of life [2]. In China, the prevalence rate of birth defects is about 56 per 1000 live births [3]. In 2020, according to the data from World Health Organization (WHO), the number of deaths from birth defects accounted for 0.38% of total deaths, reaching 36,613 cases [4]. Hunan province has a high prevalence of birth defects, with two out of every 100 newborns diagnosed with birth defects from 2005 to 2014 [5].

In order to prevent birth defects, China has implemented a three-tiered prevention strategy that aligns with its national context. The primary prevention aims to decrease the occurrence of birth defects before marriage and pregnancy. The secondary prevention focuses on reducing the occurrence of severe birth defects, primarily during the prenatal period. The tertiary prevention aims to reduce the incidence of congenital disabilities in the neonatal period [6]. According to the Law of the People's Republic of China on Maternal and Infant Health Care [7], if a fetus is diagnosed with a severe genetic disease, defect, or a disease that endangers the life or health of the pregnant woman during prenatal diagnosis, doctors are required to inform both spouses and recommend terminating the pregnancy. Therefore, the most prominent ethical issue arises in the secondary prevention of birth defects [8]. The debate over '*Should a defective fetus be born?*' has become a highly contentious issue within the domain of bioethics.

In terms of fetuses with birth defects, opinions diverge among experts. Panicola emphasizes the importance of treating fetuses with birth defects and assessing their future quality of life [9]. Clark proposes that priority treatment should be given to fetuses who are treatable or correctable [10]. Clark advocates for taking the moral criteria proposed by McCormick into consideration apart from the disease's nature and relevant circumstances [11]. Therefore, we should consider not only the fetuses’ future quality of life but also family, social, religious, cultural, emotional, and financial factors [10].

In medical ethics, the term 'rights of a pregnant woman' refers to applying clinical practices that align with her wishes [12]. Therefore, it is argued that health professionals should honor the decisions made by pregnant women [12]. In China, the Law on Maternal and Infant Health Care states that the termination of pregnancy requires explicit consent and signature from the pregnant woman. In cases where pregnant women cannot make legal decisions, their legal guardian may provide consent on their behalf [7]. The law also emphasizes that health professionals should offer medical advice before parents or other family members make the final decision. Consequently, shared decision-making between parents and other family members is recommended [13]. Shared decision-making among all family members is common in Chinese culture [14]. However, there are concerns that parents and other family members may lack medical knowledge, highlighting the need for health professionals to provide professional guidance and suggestions [12]. In Western countries, the decision-making process may involve the hospital's ethics committee and even the court [13].

Different levels and categories of medical institutions play various roles in the three-tiered prevention strategy for birth defects in China [13]. Primary care centers should screen families at high risk of birth defects and refer them to higher-level medical institutions. Prenatal diagnosis centers are generally located within provincial or municipal medical institutions, where health professionals monitor birth defect cases and provide relevant recommendations and treatment [6]. Consequently, the attitudes of these health professionals toward birth defects directly reflect the ethical challenges in the prevention and treatment of birth defects.

This study used qualitative research methods to explore the ethical attitudes of health professionals toward *'Should a defective fetus be born?'* The findings will provide empirical data and real-world evidence for formulating ethical principles for the prevention and treatment of birth defects.

## Methods

### Study design

The qualitative study used semi-structured interviews with health professionals working in maternal and child health services to explore their perspectives on birth defects. The study was reported following the Standards for Reporting Qualitative Research (SRQR) [15].

### Setting

We selected a highly-developed city (Changsha City) and a less-developed city (Huaihua City) among 14 prefecture-level cities in Hunan Province as our sampling cities based on the gross domestic product (GDP) levels [16]. A purposive sampling method was employed to recruit health professionals from three levels (primary, municipal, and provincial) of medical institutions related to maternal and child health in each city.

### Participants

We selected health professionals from various hospital levels with different professional titles who are working in various fields of maternal and child health services to maximize sample diversity. Only health professionals with working experience with fetuses and their families and those who were able and willing to be interviewed were included. Following the principle of information saturation in qualitative research [17], this study achieved saturation after recruiting 13 health professionals for interviews.

### Interview guide development

The interview guide includes general information about the health professionals and seven primary questions (see Appendix 1). These questions explore health professionals' work experiences and attitudes toward birth defects. The questions aim to address the following issues: Should a defective fetus be born? What types of fetuses with birth defects should be born? Whether the decision-making of a defective fetus should be shared among the family members.

### Data collection

Health professionals were enrolled from February 2021 to January 2022. The semi-structured interviews were conducted in Mandarin by two researchers from the study team. Both researchers had extensive knowledge of bioethics and policies related to birth defects and considerable expertise in qualitative research. The average duration of each interview was 40 minutes. One researcher posed the interview questions to the health professionals, while the other took notes and asked supplementary questions. To ensure the confidentiality of health professionals and encourage candid responses, the researchers audio-recorded the interviews after obtaining informed consent and permission from the participants. The audio recordings were transcribed by researchers, with all personal identifiers removed. The interview transcripts were uploaded to NVivo data analysis software version 12. A coding system was established using first and second-cycle coding and qualitative software, and inter-rater coding was employed to ensure reliability [18].

### Thematic analysis

Thematic analysis was conducted to analyze the data from March 2022 to May 2022. This analysis combined both deductive and inductive approaches to identify original themes [19]. Firstly, one researcher (Y.L) developed an initial structural and content code book, which included code definitions, inclusion and exclusion criteria, and examples [20]. The researcher (Y.L.) coded approximately 80% of all transcripts, which were then verified by another researcher (D.L.) to ensure agreement. Any discrepancies in the coding were resolved through discussion until a consensus was reached. The coding process yielded five themes that emerged from the interview questions. A content analysis approach was applied in the iterative process [21].

In this study, participant quotations were identified using the coding system. Each quotation was first labelled with the participant number (1–13), followed by the participant’s sex (M = male, F = female), and finally, the participant’s professional title (I = junior title, II = intermediate title, III= associate senior title, IV = senior title. For example, the 1.F.III signified a quotation from a female health professional with an associate senior title.

### Trustworthiness

The researchers referred to their field notes during the data analysis process to enhance the credibility, transferability, dependability, and confirmability of the results [22]. The interviews were peer-reviewed by the research team after being coded, and external reviews were conducted by a faculty member and a Master's student who were not part of the research team. Two health professionals were randomly selected from the participants to review the data and confirm the accuracy of the results. Purposive sampling with maximum diversity was employed to enhance the transferability of the data [20]. The report included detailed descriptions of all phases regarding the analysis process, and participants' quotations were recorded to substantiate the veracity of the findings.

## Results

### Demographic characteristics

Thirteen health professionals were interviewed, with twelve of them being female. Six professionals worked in municipal medical institutions, four in provincial medical institutions, and three in community health service centers (Table 1).

**Table 1:** Characteristics of 13 study participants

Characteristics	Value (%)
*Gender*	
Female	12 (92.31%)
Male	1 (7.69%)
*Subordinate levels and categories of medical institutions*	
Provincial maternity and child health hospital	2 (15.38%)
Provincial general hospital	2 (15.38%)
Municipal maternal and child health care hospital	4 (30.77%)
Municipal general hospital	2 (15.38%)
Community health service center	3 (23.08%)
*Professional fields*	
Health care related fields	6 (46.15%)
Genetic counseling related departments	3 (23.08%)
Obstetrics and gynecology	3 (23.08%)
Pediatric cardiac surgery	1 (7.69%)
*Professional titles*	
Junior titles	3 (23.08%)
Intermediate titles	2 (15.38%)
Associate senior titles	6 (46.15%)
Senior titles	2 (15.38%)

### Perspectives toward fetuses with birth defects

In addressing the question '*Should a defective fetus be born?*', health professionals proposed several different opinions. Five themes and 13 attributes were identified, including the severity and curability of diseases, family relations, medical assessments, social situations, and self-value orientations. A thematic analysis summary of illustrative quotations from participants is shown in Table 2.

**Table 2:** Thematic analysis summary of illustrative quotations

Theme		Illustrative quotations	
Severity and curability of the diseases	Severity of the disease and the effectiveness of treatment	2.F.III	In the case of severe fatalities and disabilities, I think it is really not possible to be born, especially for the Down’s syndrome, its intelligence level is hardly normal in anyway. It is best to intervene early when it is detected, especially if it can be done before 28 weeks. (…) Some congenital heart diseases will slowly heal themselves after birth, and some can be cured by surgery. These cases could be born. (…) Cleft lip and palate can be surgically repaired; thus, they can be born
		3.M.IV	We will tell the family that the deformity of your child is very complex
		6.F.I	The kind of malformation that is very obvious, especially the likes of Down's syndrome, I would be very sure not to have this baby born
		10.F.I	A fetus with severe congenital heart disease could not be born, if it's a mild one it could be born, the severe kind is not. (…) For those fetuses with severe fatalities and disabilities, and the fetuses that cannot be treated after birth, we should consider abandoning
		4.F.III	For the disabilities, as long as there are treatments available and medication that can be used to treat the condition, I think the birth of these fetuses are acceptable
	Impact of the disease on the child's physical function and development	13.F.II	The fetus does not grow in the womb, it is behind in gestational age and will always has intrauterine growth retardation, which can still have an impact
		4.F.III	A pterodactyl, just a small physical disability that does not affect the child to live independently
		2.F.III	The cleft lip and palate should be preserved in cases where it does not affect the child very much. There are also cases of metabolic disorders that may not be detected prenatally. However, in the case of hypothyroidism, as long as early detection and intervention is made, it can be treated as normal. (…) In the case of pterodactyl, auricular deformity, etc., it may only have some impacts on the aesthetics
		5.F.IV	Some minor deformities can be corrected after birth, or even if they are not corrected, they do not affect the quality of life or the life cycle of the child
		9.F.III	After birth with Down's syndrome, it is very difficult for the child to reach a normal level of intelligence in all areas. It's also very difficult for a child with dysgnosia to survive
Family relations	Negative impact of the pregnant woman	12.F.II	It also depends on the effects of this fetus on the pregnant woman, which could cause risk for her
	Family acceptance of the disease	9.F.III	The prerequisite is that the family is able to accept the disease and all the problems that follow from this disease, which some families cannot afford
		8.F.III	I think that there are some birth defects that can be retained, unless both families are adamant that they cannot accept the situation
		5.F.IV	The family's situation is determined by what they can afford, especially in term of the economic situation
		7.F.III	Some families do not understand these diseases and their medical knowledge is not sufficient
		1.F.III	It depends on the religious beliefs of the family, some of whom feel that they have to have the baby no matter what it is
	Value of the fetus to the family	11.F.I	In cases where pregnancy is difficult, or when the fetus is obtained through assisted reproductive technology, the fetus is precious to the family
	Negative impact of the disease on family members	3.M.IV	For some particular families, a child could be a bond between the couple so that the stability of the family can be maintained
		2.F.III	To have a child with severe Thalassemia, It can be a serious burden for the family
		10.F.I	If the healing process of the fetuses with disability is not good, it will be impossible for the family to take this burden off
		11.F.I	It is necessary to take into account the various factors of the family, the situation of family relations and whether they already have children
Medical assessments	The development of the treatment of birth defects	1.F.III	Nowadays, medical technology is developing, like the Tetralogy of Fallot, as the technology now has improved and the surgery has a 95% success rate
		4.F.III	The limitations in medical development still existed, and not all diseases are well understood
	Clinical professionals' assessment of fetal diseases and related professional advice	11.F.I	When the situation is settled in dilemma, a debate is needed. I would refer the family to a superior medical institution to seek advice from other specialists
		6.F.I	My friends have come to consult me before and I would tell them that if it was my child, I would have had it
		3.M.IV	In the situation of “should the children with birth defects be born or not?”, the medical staff is an influential factor, but not the only one
		5.F.IV	If there is any good decision that can be medically determined when will give him guidance, that will be informed as well, mainly to give him more information
Social situations	The norms and safeguards provided by society	3.M.IV	It’s good to develop some normative things in the cases of birth defects that people can enforce and are beneficial to most people. (…) I was thinking in the foreign countries, they may have a basic legal code that says induced abortion is forbidden, and national policy stipulates that it is not allowed to induce abortion or cannot be done after 24-week pregnancy, except for severe heart abnormalities. (…) In terms of religion, the Catholic Church requires that abortion is strictly forbidden, so you can't induce an abortion no matter what
		4.F.III	Without social security, it is difficult for the family to decide whether to have a child or not
	The burden of birth defects on society	1.F.III	Socially, it is also irresponsible for families to not support a child with Down's syndrome after birth, or if the family just wants to have it first, without considering the consequences
		8.F.III	Nowadays there are many people who are reluctant to have a marriage test, and I would tell them the situation of birth defect, and that it is a social responsibility for them to have the test, as well as be responsible for themselves
		10.F.I	The most important thing is that you can't have a baby if you have a serious health condition. It is a burden on the family, a burden on society, and a personal regret for the child
	The attitude of the community towards birth defects	3.M.IV	Is population quality good for a society? Is that only the healthy child could shown the society is civilized and developed? It is not clear how exactly the quality of a population is defined. (…) This society is diverse, there are tall people, short people, whose in good condition, whose with a bit of a problem, whose in good health or in poor health, that's what makes it a society, you can't say that everyone is the same
		2.F.III	Nowadays, we talk about low birth rates and low fertility rates, but for families and societies, it is still good to have a baby, not to mention the normal ones, even the baby who born with birth defects that they still has a different degree of priority
Self-value orientation	Respect for the family's decision	1.F.III	Some people feel that no matter what kind of child they have, even if it is Down's syndrome, they will still keep it. For this case, I will respect them
		3.M.IV	In the case of insisting on abortion, it is also carried out according to the personal wishes of the pregnant woman
	The individual's attitude to the birth of the fetus	3.M.IV	From my standpoint as an ordinary person, if I am not a doctor, it depends on my own view of life. Each person has their views of what they think life is. Some people think it is a sin to step on an ant, let alone to kill the fetus with birth defects. Therefore, it is really difficult to give advice without the norm, unless to advice should the fetus with birth defects be born or not?

#### Theme 1. Severity and curability of diseases

Health professionals held disparate views on the classification of birth defects, which differed according to the types and severity of defects. Some argued that fetuses with severe disabilities and malformations should be terminated, while those with minor birth defects should receive treatment. One health professional (2.F.III) suggested that fetuses with chromosomal disorders, such as Down syndrome, should not be born. However, she recommended that fetuses with conditions that could either heal on their own or be surgically repaired, such as mild congenital heart disease or cleft lip and palate, should be born. Eleven out of the 13 health professionals agreed that a fetus with a treatable disease could be born, whereas a fetus with an untreatable disease should not be carried to term.

The health professionals assessed the impact of birth defects on children’s physical function and development, including future physical function, quality of life, independence, appearance, and intelligence. One health professional (4.F.III) expressed that children with severe health problems should not be born as they might not survive and would require dependence on others to live a normal life. Furthermore, another health professional (13.F.II) emphasized the importance of examining the impact of birth defects on fetal development.

#### Theme 2. Family relations

Health professionals pointed out that the degree of acceptance of birth defects varied considerably between families. For instance, families with religious beliefs may be more accepting of a fetus with birth defects and believe that they should embrace and care for their children regardless of any imperfection. Some families may encounter difficulties in accepting the diagnosis, influenced by their relationships, financial status, and medical knowledge. One health professional (5.F.IV) suggested that families with limited financial resources might choose to terminate the pregnancy. Similarly, one health professional (7.F.III) noted that families with insufficient knowledge of fetal defects might also consider terminating the pregnancy.

Birth defects had both positive and negative effects on families. The negative impacts of a defective fetus on families included financial, caregiving, and psychological burdens. Furthermore, it could have a detrimental impact on family relationships. Conversely, viewing a fetus as a precious member of a family may bring about positive changes and growth within the family. The health professionals suggested that a fetus could reinforce family bonds and support if it were hard-won, such as through vitro fertilization, or if its mother was of advanced maternal age. Given that a pregnant woman is most closely associated with a fetus, a fetus with birth defects could negatively affect the mother’s physical and psychological health due to excessive worry about the fetus's conditions.

#### Theme 3. Medical assessments

During communication with families, health professionals provided detailed information about the specific circumstances of birth defects and offered appropriate medical advice. In cases where induced labor was clearly indicated due to a disease, the medical advice was typically consistent. Nevertheless, for controversial diseases or when counseling clients with different backgrounds, the medical advice may vary.

A primary-level health professional (11.F.I.) recommended that families should be referred to a higher-level medical institution, such as a provincial maternity hospital or a provincial children's hospital, where prenatal diagnosis could be performed. Additionally, primary-level health professionals provided medical counseling in accordance with the directions of their supervisors.

Another primary level health professional (6.F.I.) indicated that when advising friends and relatives, health professionals often provided guidance based on the presumption of 'if it were my child.' However, health professionals at provincial hospitals tended to adopt a more conservative approach. A health professional (5.F.IV) stated that she offered advice based on her expertise and medical knowledge, while another health professional (3.M.IV) emphasized that medical advice should be considered alongside other factors.

The limitations of current treatment options for birth defects were also considered. One health professional (1.F.III) believed that future medical advancements might allow for treating specific diseases, thereby rendering childbirth possible. Another health professional (4.F.III) noted the difficulty in communicating with families due to limited medical development and families' lack of knowledge about birth defects.

#### Theme 4. Social situations

The health professionals highlighted the increased demand for medical resources occasioned by fetuses with birth defects could potentially result in a rise in social burdens. Moreover, it was necessary to consider the social norms and protections provided to the affected fetuses and their families. One health professional (3.M.IV) argued that society was structured according to specific rules and norms, including religious and legal aspects, which may influence decisions on fetuses with birth defects. Clarification of laws and regulations was essential to maintain social order and safeguard the legal rights of individuals.

Another health professional (4.F.III) highlighted that birth defects had social implications that needed to be considered in terms of social security. One health professional (3.M.IV) raised concerns regarding the influence of fetuses with birth defects on the overall quality and diversity of the population. It was highlighted that the society comprised individuals with various characteristics, including those with birth defects.

#### Theme 5. Self-value orientations

The concept of 'self-value orientations' included the 'respect for families’ decision' and the 'individuals’ attitudes toward fetuses with birth defects.' One health professional (1.F.III) demonstrated a profound respect for families who maintained consistent decisions regarding fetuses with birth defects. Another health professional (3.M.IV) suggested that attitudes toward fetuses with birth defects might vary, depending on the views of health professionals or the individuals’ values. Some individuals exhibited a high degree of caution, even toward the smallest creatures, while others adopted a more indifferent stance on life and death.

### Decision-making on a defective fetus

**Fig. 1:**
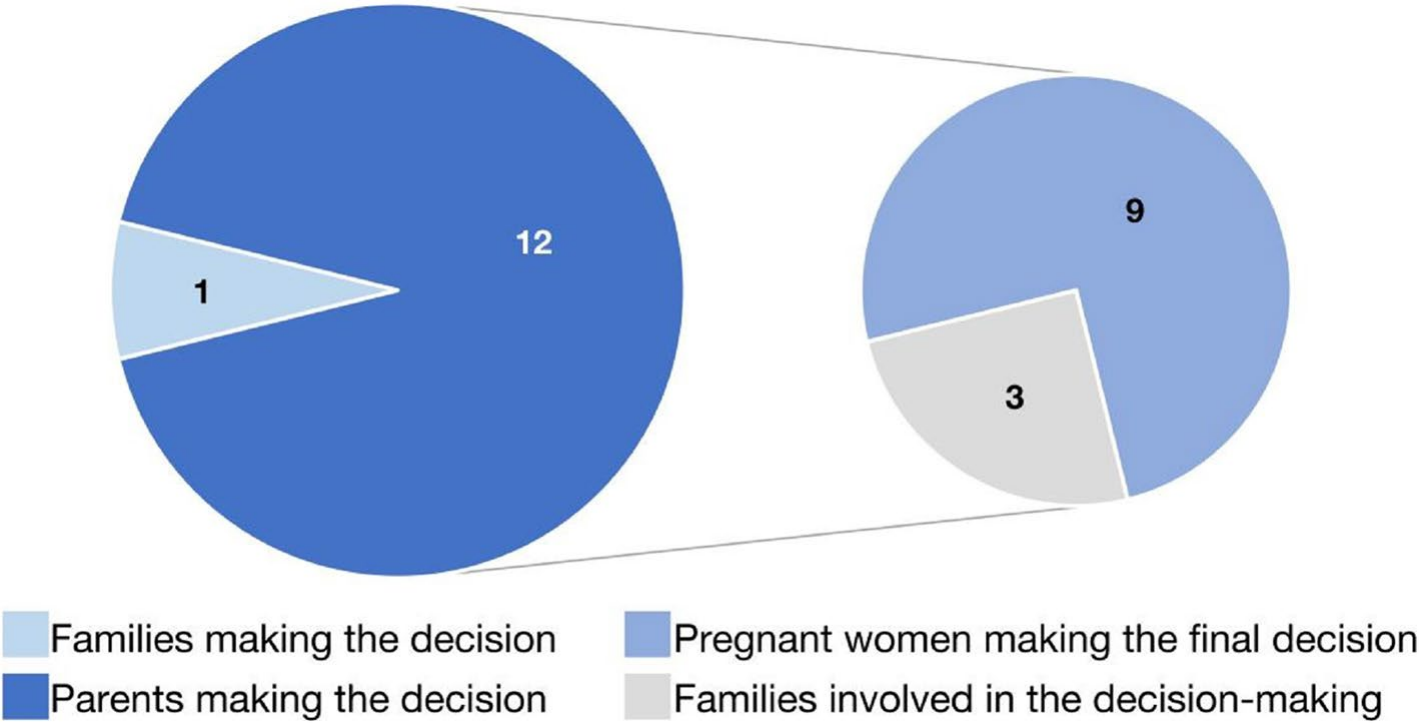
The decision-making of a defective fetus

Among the 13 health professionals interviewed, 12 expressed that parents of a defective fetus should be the primary decision-makers, while only one health professional believed that the decision should involve the entire family ( Fig. 1). Nine of the 12 health professionals indicated that pregnant women should make the final decision. Those nine health professionals emphasized that mothers had more authority than fathers in making such decisions. However, four health professionals noted the importance of considering fathers’ roles, as fathers were expected to support mothers and share caregiving responsibilities. Three health professionals reported that other family members were involved in the consent process, but parents or pregnant women ultimately made the final decision by signing the relevant documents. The interviewed health professionals believed that involving other family members was also important for decision-making on fetuses with birth defects.

Although most health professionals indicated that parents should make the final decision, all 13 health professionals reported encountering instances where the entire family ultimately made the final decision. It includes two different scenarios. In the first scenario, the parents are unable to make the decision. For example, the parents are a deaf couple unable to communicate in the usual manner. Thereby, the children's grandparents have to communicate with the doctors and make the final decision. In the second scenario, when parents are faced with the possibility of a defective fetus, they frequently require assistance from their families since the entire family shares the responsibility of raising a child with birth defects. In such cases, a respectful and influential elder within a family often makes critical decisions. The status of women within the entire family also affects decision-making. For example, women with low levels of education and those who are housewives may not have priority in decision-making, especially in rural areas.

## Discussion

Our study revealed that most health professionals believed that a fetus with a curable disease could be born, while a fetus with severe disability and teratogenesis should be terminated. This finding aligns with a Chinese study showing that fetuses with severe disability and teratogenesis might have impaired cognitive function and struggle to live fulfilling lives [8]. Savulescu also argued that children with birth defects might endure a life of constant pain, limited movement and sensation, and impaired social function, which could be worse than death [23]. The WHO Report on Ethical Issues in Medical Genetics further supports the timely termination of pregnancy in cases involving severely disabling and teratogenic fetuses as a measure in line with medical ethics [24]. Furthermore, Savulescu acknowledges that mild to moderate intellectual disability may be a distinct condition [23]. Consequently, the decision to bring children with such a disability into the world should depend on whether their lives would be worth living.

In the field of birth defect prevention and treatment, health professionals often encounter situations where the condition of a fetus with birth defects cannot be accurately diagnosed due to limitations in current medical technologies. Additionally, assessing the prognosis of a fetus with birth defects, including its potential for recovery, can be challenging. Consequently, health professionals must consider multiple factors before providing counseling to parents on whether or not the fetus should be born. These considerations include the nature of the fetus's illness, the family’s circumstances, and various medical and social factors.

It is, therefore, imperative to establish ethical norms related to birth defects to guide health professionals in delivering ethically sound services. Additionally, it is recommended that ethical training for health professionals engaged in the field of birth defect prevention and treatment should be enhanced. For medical institutions qualified for prenatal diagnosis, it is of utmost importance to reinforce the construction of ethics committees. To address the ethical issues in this field, it is recommended that prenatal diagnosis ethics committees be established in medical institutions and that the specific circumstances of each institution be considered. When health professionals encounter cases with evident ethical implications, it is essential to have appropriate responses and establish broader ethical norms or systems within prenatal diagnosis facilities. Medical institutions should refer cases that raise significant ethical concerns to their ethics committees for review. Such cases include instances where doctors consider the fetus viable, yet a woman and her family desire an abortion, or the converse.

Regarding diseases of fetuses, the interviewed health professionals believed that the severity and curability of diseases should be considered. This perspective is supported by Clark and Lu et al. [10, 25]. In determining whether a fetus with birth defects should be carried to term, it is necessary to consider factors such as the fetus's self-awareness and future ability to live independently. Family relations are also an important consideration. The occurrence of birth defects has a significant impact on families [26]. In addition to the financial burden attributable to diseases [27], the psychological burden on family members should also be considered [28]. Therefore, it is crucial to acknowledge the impact of diseases on families. Given that a fetus with birth defects will become a family member after birth, it is essential to consider the treatment and attitudes of families toward the fetus. The findings indicate that the efficacy of treatment and recovery for a fetus with birth defects is closely related to families’ involvement and support. The level of support provided by families is contingent upon the value placed upon fetuses, which ultimately affects the effectiveness of post-birth treatment [27]. Additionally, when a fetal disease poses a significant risk to a pregnant woman's life, it is recommended that her interests should be prioritized in decision-making [29].

The interviewed health professionals highlighted two medical factors: advancements in medical technologies and advice provided to families regarding birth defects. Over the years, numerous birth defects have been effectively treated and managed, enabling individuals to live normal lives [30]. However, certain complex and unpredictable conditions still pose challenges for health professionals in terms of diagnosis and prognosis due to the limitations of available diagnostic techniques. Nevertheless, it is of utmost importance that those working in medicine have a comprehensive understanding of the diseases in question to provide the best possible support to the affected families [27]. Therefore, the capacity of health professionals who are furnished with precise and comprehensive medical counseling expertise also influences the decision on a fetus's birth [12]. Despite clear principles of medical ethics regarding providing information about healthcare, there are still instances of insufficient or uninformed information in practice [12, 24]. To address this, the interviewed health professionals proposed the development of more precise and effective principles or procedures for delivering medical information in healthcare. This can ensure that parents and families are better informed and have access to accurate medical resources. Furthermore, it is necessary to pay attention to the ethics committees in medical institutions. Such committees can assist health professionals when they are uncertain about the diagnosis of birth defects and provide timely support to families in need.

From a sociological perspective, the interviewed health professionals considered whether society could protect families with fetuses with birth defects to alleviate the burden on families. Others also contemplated whether the birth of a fetus with birth defects would be a burden to society. Moreover, the long-term impact of birth defects on the offspring's physical development could affect society's overall health. Some studies have presented a different viewpoint, arguing that such a perspective disregards the life of a fetus and is inconsistent with the principles of medical treatment and fundamental ethical principles [24]. In 2021, China implemented a policy promoting eugenics, emphasizing the importance of preventing and treating birth defects to improve the overall population health quality and promote healthy and balanced development of our society [31]. It is also important to note that this policy may potentially lead to increased pressure and discrimination against individuals with disabilities and genetic disease carriers. It is crucial to consider the differing attitudes of the general public toward fetuses with birth defects, whether these are inclusive or exclusive. The attitudes of the general public toward fetuses with birth defects vary between Western and Eastern countries due to differences in social-cultural contexts and individual values [32]. Western countries emphasize individualism and self-actualization, while Eastern countries, influenced by Confucianism culture, prioritize collectivism and familial values [33]. Consequently, when addressing birth defects, individuals in Eastern countries prioritize societal benefits, considering factors such as population quality and social diversity. It is recommended that health professionals consider both individual and societal perspectives comprehensively.

Most of the 13 health professionals believed that parents should make the decision on birth defects. However, a minority argued that pregnant women or families should decide together. This perspective aligns with a Chinese study [25]. However, it contradicts the principle of medical ethics, which emphasizes respecting mothers’ reproductive rights [24]. The complexity of the situation surrounding a fetus with birth defects necessitates a collaborative decision-making process involving parents and other family members, particularly in comparison to the birth of a normal fetus.

In examining real-world cases, all health professionals acknowledged experiencing situations where families played a role in making the final decision. Nevertheless, seven health professionals indicated that, in practice, they would insist on obtaining the parents' signatures on the decision. In certain situations, other family members bear the responsibility of making decisions regarding a fetus with birth defects. This can occur when parents cannot decide by themselves or authorize a family member to decide on their behalf. Factors such as financial constraints and reliance on family support can influence families’ decision-making process. Due to traditional Chinese culture, it is customary for family members to share decision-making about important events. Some studies propose that families should be a fundamental unit for reproductive decisions [34] and that Chinese families always raise their children through joint participation in their care and upbringing [14]. Similarly, in clinical medical decisions, it is common for family members to make the decision collectively [14, 35]. Therefore, when a family is faced with a decision, grandparents or other family members are involved in the decision-making process.

It is important to recognize the roles of pregnant women in the pregnancy process, as they have primary experience. According to the interviewed health professionals, the status of pregnant women plays a significant role in their decision-making and reproductive rights. Some health professionals argued that some pregnant women faced disadvantages due to their family status, while some believed that pregnancy could lead to an increased sense of independence and improved family status of the women. Consequently, the concept of shared decision-making not only encompasses the idea of relational autonomy but also serves to balance the relationships among women, family members, society, and authority represented by doctors and national fertility policies [36]. Medical knowledge can also assist families in reaching a consensus regarding the decision about a fetus. Furthermore, the provision of social protection policies or clear guidelines on the prevention and treatment of birth defects can assist families in their decision-making process and alleviate the potential burden of caring for a fetus with birth defects. Similarly, in Western countries, an ethics committee is involved in the decision-making process [13]. Medical institutions could enhance the establishment of ethics committees, including specific committees for prenatal diagnosis, especially in qualified prenatal diagnostic institutions, to assist families in making decisions.

This study has some limitations. Firstly, the study solely explored the question of whether fetuses with birth defects should be born without addressing the ethical issue of whether these fetuses have the same rights to live as normal fetuses. Secondly, the study was conducted with professionals, possibly leading to a social expectation bias. Health professionals frequently adopt a medical practitioner's perspective and prioritize the principle of saving lives when addressing these questions. This study prompted the question of *'Should a defective fetus be born?'.* However, the broader ethical considerations related to birth defects remain to be addressed.

## Conclusions

It is important to consider the input of health professionals who are in a position to address several related issues, including diseases, family dynamics, medical aspects, societal factors, and self-value orientations. While many health professionals believed that the decision-making regarding birth defects should primarily rest with pregnant women and then involved a fetus's father and other family members, real-world cases often present more complex scenarios. Hence, health professionals should be furnished with standard norms or policies regarding birth defects to offer informed suggestions. It is crucial to establish ethics committees, including a committee on prenatal diagnosis, to address current ethical issues in the prevention and treatment of birth defects. Furthermore, it is of paramount importance to provide families affected by birth defects with both medical knowledge and societal support.

### Supplementary Information

Supplementary Material 1: Table S1. Semi-structured interview guide.

## Data Availability

The raw data supporting the conclusions of this article will be made available by the authors without undue reservation.

## Abbreviations

WHO: World Health Organization
SRQR: Standards for Reporting Qualitative Research

## References

[1] World Health Organization. Congenital disorders 2023 [Available from: https://www.who.int/news-room/fact-sheets/detail/birth-defects.

[2] National Institutes of Health, Eunice Kennedy Shriver National Institute of Child Health and Human Development. Birth Defects 2020 [Available from: https://www.nichd.nih.gov/health/topics/birthdefects.

[3] Xie D, Xiang Y, Wang A, Xiong L, Kong F, Liu Z (2020). The risk factors of adverse pregnancy outcome for pre-pregnancy couples in Hunan, China: a cross-sectional study based on population. Medicine (Baltimore).

[4] World Health Organization. China: Congenital anomalies 2020 [Available from: https://www.worldlifeexpectancy.com/china-congenital-anomalies.

[5] Xie D, Yang T, Liu Z, Wang H (2016). Epidemiology of birth defects based on a birth defect surveillance system from 2005 to 2014 in Hunan Province, China. PloS one..

[6] National Health Commission of the People's Republic of China. National plan for the Comprehensive Prevention and control of birth defects in China. 2018.

[7] Standing Committee of the National People's Congress. Law of the People's Republic of China on Maternal and Infant Health Care (Revised in 2017). In: Congress SCotNPs, editor.: Standing Committee of the National People's Congress; 2017.

[8] Jiang S (2009). Ethical Thinking on Intervention of Birth Defects (in Chinese). Med Philos (A)..

[9] Panicola MR. Catholic teaching on prolonging life: setting the record straight. 9781589012424: Georgetown University Press; 2007. p. 29–51. 12945451

[10] Clark PA. Decision-making in neonatology : an ethical analysis from the catholic perspective. IntechOpen; 2012.

[11] McCormick RA (1989). Theology and Bioethics. Hastings Cent Rep.

[12] Chervenak FA, McCullough LB (2017). Ethical dimensions of the fetus as a patient. Best Pract Res Clin Obstet Gynaecol.

[13] Ott BB, Olson RM (2011). Ethical issues of medical missions: the clinicians' view. HEC Forum.

[14] Lin ML, Pang MC, Chen CH (2013). Family as a whole: elective surgery patients' perception of the meaning of family involvement in decision making. J Clin Nurs.

[15] O'Brien BC, Harris IB, Beckman TJ, Reed DA, Cook DA (2014). Standards for reporting qualitative research: a synthesis of recommendations. Acad Med.

[16] The People’s Government of Hunan Province. Statistical Communiqué on Hunan's Economic and Social Development in 2020. In: Hunan Provincial People's Government, editor. 2021.

[17] Given LM (2015). 100 Questions (and Answers) about Qualitative Research.

[18] Morse JM (2015). Critical analysis of strategies for determining rigor in qualitative inquiry. Qual Health Res.

[19] Carcary M. Advancing the research audit trail: A ten year retrospective. Kidmore End: Academic Conferences International Limited. 2020

[20] Miles MB. Qualitative data analysis a methods sourcebook. 4th ed. SAGE Publishing; 2019.

[21] Bengtsson M (2016). How to plan and perform a qualitative study using content analysis. NursingPlus open.

[22] Simula BL. The coding manual for qualitative researchers. 3rd ed. SAGE; 2018. p. 173–5.

[23] Savulescu J (2002). Is there a "right not to be born"? Reproductive decision making, options and the right to information. J Med Ethics.

[24] Wertz DC, Fletcher GF, Berg K & WHO Human Genetics Programme. Review of ethical issues in medical genetics : report of consultants to WHO. World Health Organization. 2003.

[25] Lu Y, Zhang J (2008). Give up or cure affect the moral baseline: ethics thinking about curing the defective infants or not (in Chinese). Med Philosophy(B)..

[26] Awoyale T, Onajole AT, Ogunnowo BE, Adeyemo WL, Wanyonyi KL, Butali A (2016). Quality of life of family caregivers of children with orofacial clefts in Nigeria: a mixed-method study. Oral Dis.

[27] Song X, Li N, Liu J, Chen G, Zhang L, Li C (2012). Depression and its influencing factors among mothers of children with birth defects in China. Matern Child Health J.

[28] Yilmaz G, Küçük AD (2021). Evaluation of care burden among mothers of children with a disability: Correlation between physical activity, quality of life, and sleep quality: a cross-sectional study. Perspect Psychiatr Care.

[29] Shah NR, Kim KM, Wong V, Cohen E, Rosenbaum S, Cahan EM (2021). Mothers of children with major congenital anomalies have increased health care utilization over a 20-year post-birth time horizon. PloS one..

[30] Walani SR, Penny N, Nakku D (2023). The global challenges of surgical congenital anomalies: evidence, models, and lessons. Semin Pediatr Surg.

[31] The Communist Party of China Central Committee. Decision of the Central Committee of the CPC and the State Council on Optimizing the Family Plannino Policy to Promote Long-term and Balanced Development of the Population (in Chinese). In: The Communist Party of China Central Committee and The State Council. the Xinhua News Agency. 2021.

[32] Hofstede G, Draguns JG. Culture's consequences: comparing values, behaviors, institutions, and organizations across nations. 2007. p. 43–58.

[33] Cohen AB, Wu MS, Miller J (2016). Religion and culture: individualism and collectivism in the east and west. J Cross Cult Psychol.

[34] Lin ML, Huang CT, Chen CH (2017). Reasons for family involvement in elective surgical decision-making in Taiwan: a qualitative study. J Clin Nurs.

[35] Ma D, Zuo M, Liu L (2021). The information needs of Chinese family members of cancer patients in the online health community: what and why?. Inf Process Manage.

[36] Dong D, Ahmed S, Nichini E, Yi H, Jafri H, Rashid Y (2021). Decision making on antenatal screening results: A comparative Q-method study of women from two Chinese cities. Health Expect.

