# Supplementary material for: What are they considering when they face a fetus with birth defects? A qualitative study on ethical attitudes of health professionals in China
Source: Glob Health Res Policy. 2024 Jul 15;9:27. doi: 10.1186/s41256-024-00370-1 (PMC11247724; doi:10.1186/s41256-024-00370-1)
Supplement: Supplementary file 1 — Supplementary Material 1: Table S1. Semi-structured interview guide. [file 41256_2024_370_MOESM1_ESM.docx]

**Supplementary material**

TableS1 Semi-structured interview guide

| **Semi-structured interview guide** |
| --- |
| 1. Can you describe the most impressive birth defection-related case you have ever encountered in your work (or any unforgettable experience you have had while providing maternal and child health services for a fetus with birth defects)? |
| - How was a fetus with birth defects detected? |
| - How did you offer advice? |
| - What was the result? |
| - In addition to giving professional medical advice, when would you give more explicit or direct advice? |
| 2. Under what circumstances do you think a fetus with birth defects should be born or should be aborted? |
| - How do you consider a fetus with birth defects? |
| - Any consideration? |
| 3. How do you consider who should make the decision whether a fetus with birth defects should be born or aborted?   - Any reasons? |
| - How do you understand the meaning of decision-making? |
| 4. How are decisions often made in real-life cases? |
| - In real-life cases, do the decisions made differ from what you think? What do you do in such situations? |
| 5. What do you think about birth defects? |
| - How would you explain birth defects to a pregnant woman or family member? |
| - What do you pay attention to when "informing" in such a situation? |
| 6. How do people around you think about a fetus with birth defects? |
| 7. Do you know whether there are any services available for a fetus with birth defects? |
| - What kind of help (or services) do they need most? Any reasons? |
